# Therapeutic effects of IL-33/ST-2 pathway inhibition combined with albendazole on hepatic fibrosis and immune regulation in alveolar echinococcosis: in vivo and in vitro evidence

**DOI:** 10.1186/s13071-026-07355-8

**Published:** 2026-04-03

**Authors:** Shi-Lei Cheng, Xiu-Mei Ma, Bin-Jie Wu, Yu-Xuan Yang, Hai-Ning Fan

**Affiliations:** 1 Department of Hepato-Pancreatobiliary Surgery, The Research Key Laboratory for Echinococcosis of Qinghai Province, Qinghai University Affiliated Hospital, No. 29 of Tongren Road, Chengxi District, Xining, 810001 Qinghai Province China; 2 Research Center for High Altitude Medicine, Key Laboratory of High Altitude Medicine (Ministry of Education), Qinghai University Medical College, Xining, 810001 Qinghai Province China; 3 Center of Molecular Pathology, Qinghai Provincial People’s Hospital, Xining, 810001 Qinghai Province China; 4 Department of Infection Control, Qinghai Branch Hospital of the First Affiliated Hospital of Tianjin University of Traditional Chinese Medicine, Xining, 810001 Qinghai Province China

**Keywords:** Albendazole, Echinococcosis, Eosinophils, IL-33, Immune system

## Abstract

**Background:**

This study aimed to examine the role of the interleukin-33 (IL-33)/suppression of tumorigenicity 2 (ST-2) signaling pathway in hepatic fibrogenesis within the microenvironment of alveolar echinococcosis (AE). The therapeutic efficacy and immunomodulatory effects of concurrent IL-33/ST-2 pathway inhibition and albendazole (ABZ) treatment were also evaluated.

**Methods:**

Protein expression levels of IL-33 and ST-2 in liver were examined in a murine model of AE using immunohistochemistry. Flow cytometry was used to detect the expression of IL-33 and ST-2 on eosinophils in the blood, liver, and spleen, respectively. Phagocytosis assays, migration assays, and western blot analysis were performed to investigate the effects of IL-33 and ST-2 on eosinophils and hepatic stellate cells, so as to evaluate their roles in hepatic fibrosis and eosinophil activity. Following targeted suppression of the IL-33/ST-2 signaling pathway in combination with ABZ administration, hepatic fibrosis in liver and therapeutic outcomes were assessed through Masson staining, western blot analysis, and liver index measurements. Immune function was assessed via spleen index evaluation and enzyme-linked immunosorbent assay in blood, while hepatic function was assessed by measuring serum alanine aminotransferase and aspartate aminotransferase levels.

**Results:**

The AE model demonstrated elevated expression of IL-33 and ST-2 in hepatic tissues. In vitro analyses indicated that IL-33 and ST-2 promoted profibrotic phenotypes, including upregulation of α-smooth muscle actin (α-SMA), in hepatic stellate cells, supporting their role in fibrosis development, and modulated eosinophil activity. Inhibition of IL-33/ST-2 expression, followed by ABZ administration, enhanced therapeutic efficacy, improved liver function parameters, and modulated immune responses in AE mice. Combined therapy led to superior outcomes compared with monotherapy, with evidence of reduced hepatic injury and restored immunological homeostasis.

**Conclusions:**

The IL-33/ST-2 signaling pathway contributes to the pathogenesis of hepatic fibrosis and immunological dysregulation in AE by influencing eosinophil function. Combined intervention targeting this pathway and albendazole administration confers enhanced therapeutic efficacy for AE, encompassing antifibrotic action, liver function recovery, and immune modulation.

**Graphical Abstract:**

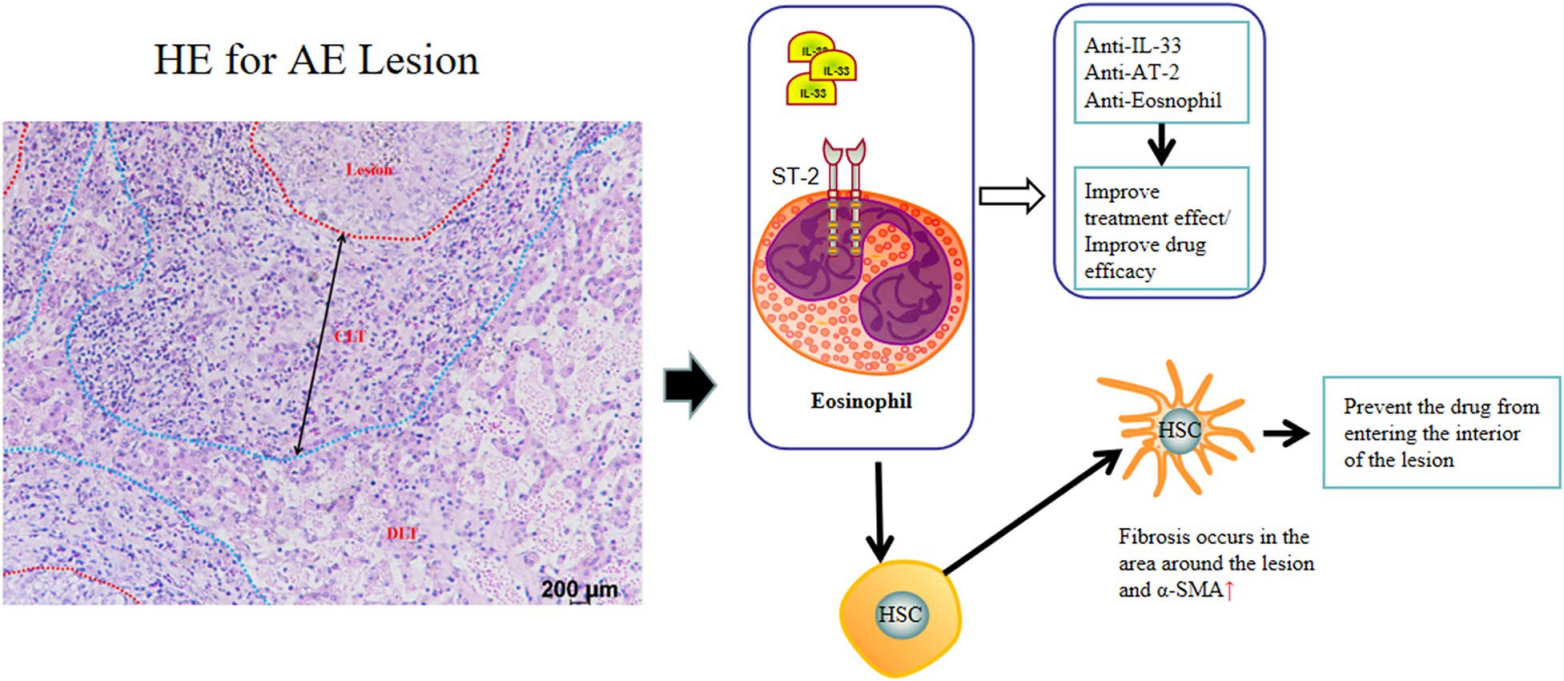

**Supplementary Information:**

The online version contains supplementary material available at 10.1186/s13071-026-07355-8.

## Background

Echinococcosis, also referred to as hydatid disease, is a zoonotic parasitic infection caused by the larval stages of cestodes of the genus *Echinococcus*, which primarily invades the liver and lungs of humans and animals [1–3]. While *Echinococcus granulosus* is responsible for cystic echinococcosis (CE), alveolar echinococcosis (AE) is caused by *Echinococcus multilocularis*. Cystic echinococcosis (CE) is endemic in many regions, with high prevalence in China, Central Asia, Russia, Europe, and North America, whereas AE predominantly occurs in the Northern Hemisphere [4, 5]. Surgical intervention remains the primary therapeutic approach for CE and AE. In AE, surgery aims to achieve radical excision of parasitic tissue, whereas in CE, surgical management aims to achieve complete removal of lesions to prolong survival and enhance quality of life [6–9]. Benzimidazole compounds are commonly used as adjunct pharmacologic agents following surgery; however, their clinical efficacy remains limited.

Albendazole (ABZ), the principal antiparasitic drug used in the management of echinococcosis, demonstrates poor oral bioavailability (< 5%) owing to its low aqueous solubility (0.07 μg/mL at 37 °C) and extensive first-pass metabolism [10, 11]. More critically for AE, one of the principal factors limiting drug efficacy is the presence of a dense, avascular, and highly fibrotic capsule surrounding parasitic lesions. This unique pseudotumoral structure acts as a physical barrier, severely restricting drug penetration and preventing adequate delivery of the therapeutic compound to the metacestode (larval stage) and protoscoleces. This fibrotic response is not merely a passive host defense but an active pathological process primarily driven by the activation of hepatic stellate cells (HSCs), leading to the dysregulation of extracellular matrix (ECM) synthesis and degradation, and excessive ECM proliferation and deposition [12, 13].

Previous studies have indicated high expression of interleukin-33 (IL-33) during hepatic fibrogenesis, with fibrosis regression correlating with reduced IL-33 levels [14]. Moreover, IL-33 has been implicated in promoting collagen fiber synthesis, identifying it as a key contributor to fibrotic progression [15–17]. The IL-33/ST-2 (suppression of tumorigenicity 2) axis is therefore perfectly positioned at the intersection of immunity and fibrosis. Its receptor, suppression of ST-2, is expressed predominantly on both immune effectors and structural cells. Notably, it is expressed on key innate immune cells like eosinophils and [18–20], critically, also on the very cells responsible for fibrosis—HSCs [21].

This dual expression profile provides a compelling mechanistic rationale for our study design. We hypothesize that a functional cross-talk exists wherein IL-33, potentially released by activated eosinophils or other damaged cells within the lesion, binds to ST-2 receptors on HSCs. This interaction could directly promote HSC activation and α-smooth muscle actin (α-SMA) expression, thereby fueling the fibrotic cascade that forms the drug-impenetrable capsule. Conversely, IL-33 signaling in eosinophils may also influence their recruitment, survival, and effector functions, potentially creating a feed-forward loop that sustains both inflammation and fibrosis.

The present study focused on AE and aimed to investigate the role of eosinophils in a murine model of AE and to elucidate the regulatory effects of the IL‑33/ST‑2 signaling pathway on eosinophil function. By establishing how IL-33/ST-2 governs eosinophil behavior and how it directly impacts HSC-driven fibrosis, we sought to define the molecular basis of this pathological cross-talk. In addition, IL-33/ST-2 pathway inhibition was employed to disrupt the fibrotic capsule surrounding hydatid lesions, followed by ABZ treatment, to determine whether pathway modulation could enhance drug penetration and thereby improve therapeutic efficacy.

## Methods

### Experimental design overview

To systematically investigate the role of the IL-33/ST-2 axis in AE pathogenesis and its impact on ABZ efficacy, this study was conceived as a multi-tiered investigation, progressing logically from in vivo observation to in vitro mechanistic dissection and finally returning to in vivo therapeutic validation. (A) Initial in vivo phenotypic analysis: In an established AE mouse model, we first performed a comprehensive profiling of the IL-33/ST-2 axis. This included measuring its expression in the blood, lymphatic system, and liver, analyzing its cellular distribution among immune cells and quantifying its levels within the lesion microenvironment. (B) Mechanistic in vitro dissection: To pinpoint the cellular mechanisms identified in vivo, we conducted two sets of in vitro experiments: (1) treating eosinophils with IL-33 to assess its impact on their phagocytic activity and migratory capacity and (2) treating hepatic stellate cells (HSCs) with IL-33 to evaluate its direct effect on the expression of the activation marker α-SMA. (C) Translational in vivo therapeutic validation: Guided by the established mechanism, we proceeded to the final stage of our study: evaluating the therapeutic efficacy of combining IL-33 pathway intervention with ABZ treatment in the AE model. The efficacy was comprehensively assessed through pathological, immunological, and liver function analyses.

### Materials and experimental animals

#### Isolation of *E. multilocularis* protoscoleces and acquisition of *E. multilocularis* antigen

Lesions of *Echinococcus multilocularis* were obtained from Mongolian gerbils infected with *E. multilocularis* and maintained in our laboratory. Animals were euthanized following anesthesia: isoflurane inhalation anesthesia was first administered (concentration: 1.5–2%, continuous inhalation for 2–3 min to ensure adequate anesthesia prior to the procedure), after which euthanasia was performed by cervical dislocation (resulting in neck fracture) to minimize suffering of the experimental animals.

*E. multilocularis* protoscoleces (PSCs) were isolated as described previously [22]. Metacestode tissues were isolated from the abdominal cavity and repeatedly after thorough washing with phosphate-buffered saline (PBS) to remove blood and fluid from the cyst surface. The cleaned lesions were cut into small pieces in pre-cooled physiological saline, then sequentially passed through sterile filters with pore sizes of 100 μm and 40 μm to collect protoscoleces. During filtration, physiological saline was continuously used for rinsing (lesions and protoscoleces retained on the filters were collected separately for antigen preparation). Finally, naturally settled protoscoleces were obtained. Viability was assessed and protoscoleces were counted using the Trypan blue exclusion assay, after which they were cultured in RPMI‑1640 medium (cat. no. PM150110A, Procell Life Science & Technology Co., Ltd., Wuhan, Hubei, China) supplemented with 15% fetal bovine serum (FBS) (cat. no. A5669701, Thermo Fisher, MA, USA).

The remaining collected lesions and protoscoleces were homogenized using a tissue homogenizer (model OSE‑Y50, TIANGEN Biotech, Beijing, China), followed by centrifugation at 4 °C and 12,000 rpm for 20 min. The supernatant was filtered through a sterile filter (pore size 0.22 μm; cat. no. SLGP033RB, Merck Millipore, Darmstadt, Germany), and the protein concentration was adjusted to 20 mg/mL using a BCA protein assay kit (cat. no. A55860, Thermo Fisher, MA, USA) and stored at −80 °C. The prepared *Echinococcus multilocularis* antigen (E.m‑Ag) and protoscoleces were used for in vitro intervention experiments and for establishing an *E. multilocularis* infection model.

#### Establishment and validation of the echinococcosis model

BALB/c mice (6–8 weeks old, weighing 30–40 g) were obtained from the Laboratory Animal Center of Xi’an Jiaotong University (Xi’an, China). The procedure was as follow: transfer the PSCs from the culture dish into a 15-mL centrifuge tube, wash them repeatedly three times with sterile PBS, and allow them to settle naturally to remove suspended dead or low-vitality PSCs as well as residual culture medium. Meanwhile, take a small aliquot of the well-mixed PSCs for Trypan blue staining, observe their morphology and viability under the microscope, and perform cell counting. Only PSCs with a viability ≥ 90% were used for animal inoculation. A total of 25 BALB/c mice were assigned to the infection group and the murine echinococcosis model was established by inoculating PSCs (1500 viable PSCs suspended in sterile 0.9% sodium chloride (NaCl) through ultrasound-guided hepatic puncture using a diagnostic ultrasound system (ZS3, Shenzhen Mindray Biomedical Electronics Co., Ltd., China)). The control group received an equivalent volume of physiological saline. All animals were housed under specific pathogen-free (SPF) conditions with ad libitum access to standard laboratory diet and water. After approximately 1 month of feeding, hepatic lesion development was monitored using small-animal ultrasonography (ZS3, Shenzhen Mindray Biomedical Electronics Co., Ltd., China) to confirm model establishment. Upon successful verification of hepatic infection, mice were enrolled in experimental treatment protocols.

### In vivo validation of IL-33 and ST-2 expression in the AE model

#### Immunohistochemical analysis of IL-33 and ST-2 in hepatic tissue

Mice confirmed to be infected with *Echinococcus* were euthanized, and liver tissues were collected. The liver samples were fixed in 4% formaldehyde, dehydrated, embedded in paraffin, and sectioned at a thickness of 3 μm. The sections were dewaxed and rehydrated following incubation at 60 °C for 30–45 min (xylene I and II, 15 min each; anhydrous ethanol I and II, 5 min each; 95%, 90%, and 80% ethanol I and II, 5 min each). Antigen retrieval was performed using 0.5 M EDTA buffer (pH 8.0) under high pressure conditions for 10–15 min. After outlining the tissue region, endogenous peroxidase activity was quenched, followed by blocking of a nonspecific binding using either serum or 5% skim milk at 37 °C for 10 min. The sections were then incubated overnight at 4 °C with the primary antibodies, followed by incubation with the secondary antibody at 37 °C for 1 h, followed by treatment with the streptavidin–biotin–peroxidase complex for 10 min. Between each step, slides were washed three times with PBS (3 min per wash). Color development was achieved using 3,3′-diaminobenzidine (DAB) chromogenic reagent, and nuclei were counterstained with hematoxylin. The staining duration was adjusted under microscopic observation to achieve optimal contrast. Blue coloration was obtained by rinsing in running water. The sections were mounted using neutral resin, and microscopic images were captured for analysis. Images were acquired using the TissueGnostic Analysis System (version 7.1; StrataQuest, Austria, https://tissuegnostics.com) under identical exposure conditions. This system can directly identify negative and positive cells, marking them in green and red, respectively, and perform automatic counting. The primary antibodies used for immunohistochemical detection included anti-ST-2 antibody (ab194113, dilution 1:2500, Abcam, Cambridge, UK) and anti-IL-33 antibody (ab187060, dilution 1:1000, Abcam, Cambridge, UK).

#### Flow cytometry detection of IL-33 and ST-2 in lymphocytes and eosinophils

To assess IL-33 and ST-2 expression in immune cell populations, including lymphocytes and eosinophils, flow cytometry was performed. Approximately 0.1 g of hepatic and splenic tissue from each mouse was homogenized in 250 μL of PBS using a sterile grinding rod. The suspension was filtered through a 200-mesh gauze into chilled flow cytometry tubes. A total of 200 μL of anticoagulated whole blood, hepatic, and splenic tissue suspensions were added to flow cytometry tube and mixed thoroughly. After washing with 1000 μL of PBS, the cell suspension was centrifuged at 450*g* and the supernatant was discarded. Red blood cell lysis buffer was then added, and lysis was performed on ice for 15 min with gentle agitation three times during incubation. Cell surface markers CD45, CCR3, and Siglec-F were stained at 37 °C for 30 min. Following PBS washing, cell membranes were permeabilized for 20 min, and intracellular IL-33 and ST-2 antibodies were added for staining. The stained cells were analyzed using a multicolor flow cytometer (BD FACSCelesta, Becton, Dickinson and Company, Franklin Lakes, NJ, USA).

Gating strategy (Supplementary Fig. S1): Singlets were identified on the basis of forward and side scatter characteristics. CD45^+^ events were defined as leukocytes. Eosinophils were identified as the Siglec-F^+^CCR3^+^ subpopulation within the CD45^+^ gate. The reagents used included 10 × red blood cell (RBC) lysis buffer (ab204733, Abcam, Cambridge, UK) and an intracellular fixation and permeabilization buffer set (product no. eBioscience-88-8824-00, Thermo Fisher Scientific, Waltham, MA, USA). The following antibodies were used: APC anti-mouse CD45 recombinant antibody (BioLegend, cat. no. 157606, San Diego, CA, USA), PerCP/Cyanine 5.5 anti-mouse CD193 (CCR3) antibody (BioLegend, cat. no. 144516, San Diego, CA, USA), FITC anti-mouse CD170 (Siglec-F) antibody (BioLegend, cat. no. 155504, San Diego, CA, USA), Brilliant Violet 421^™^ anti-mouse IL-33Rα (IL1RL1, ST-2) antibody (BioLegend, cat. no. 145309, San Diego, CA, USA), and PE anti-IL-33 antibody (ab282176, Abcam, Cambridge, UK).

Through in vivo experiments, we first clarified the expression patterns of IL‑33 and ST‑2 in the AE model and their relationship with eosinophils; subsequently, we further conducted in vitro experiments to validate the specific mechanisms of action of IL‑33 and ST‑2.

### In vitro validation of IL-33 and ST-2 function

#### Effect of IL-33 on eosinophil phagocytic activity

Eosinophilic leukemia cells (EOL-1) (5 × 10^5^ cells/mL) were cultured in RPMI-1640 (cat. no. PM150110A, Procell Life Science & Technology Co., Ltd., Wuhan, Hubei, China) complete medium until stabilization. Then, the cells were divided into three groups, with five wells (or dishes) per group, following a 48-h pre-incubation with specific stimulating agents. Prior to the assay, cells were counted and incubated with fluorescently labeled *Escherichia coli* (green fluorescence, dilution 1:500) for 2 h on a shaker. After incubation, cells were centrifuged and washed with PBS to remove unbound bacteria. Flow cytometry (FACSCelesta, Becton, Dickinson & Co., NJ, USA) was used to assess the phagocytic activity of eosinophils. The experimental conditions included stimulation with recombinant human IL-33 protein (active) (3 ng/mL, ab281811, Abcam, Cambridge, UK), E.m-Ag, and PBS as a negative control. This experiment was independently repeated three times.

#### Effect of IL-33 on eosinophil migration

EOL-1 cells were cultured in RPMI-1640 complete medium (supplemented with 10% fetal bovine serum, 100 U/mL penicillin, and 100 μg/mL streptomycin). For the Transwell migration assay [23, 24], the cells were harvested, washed twice with PBS, and resuspended in serum-free RPMI-1640 medium. In addition, the cell suspension was counted using a hemocytometer, and the density was adjusted to 1 × 10^5^ cells/mL before being transferred into the upper chamber of 24 Transwell plates (cat. no. 140620, Thermo Fisher, USA), each well containing 450 μL of the cell suspension. The lower chambers were filled with complete medium supplemented with specific chemoattractants. The experiment included the following groups: blank control (PBS, 50 μL/mL; *n* = 5), E.m-Ag (20 mg/mL; *n* = 5), and IL-33 (3 ng/mL; ab281811, Abcam, Cambridge, UK) treatment groups (*n* = 5). Following a 24-h incubation in a humidified 5% CO_2_ incubator (MCO-20AIC, SANYO, Osaka, Japan), cells that migrated to the lower chamber were collected and centrifuged at 1000 rpm for 5 min. After discarding the supernatant, PBS was added and the cells were stained with 4,6-diamidino-2-phenylindole (DAPI). The cell suspension (100 μL) was spread on slides, and eosinophil migration was quantified and imaged using fluorescence microscopy (Cytation5, Biotek, Winooski, USA). The number of eosinophils in five random fields per insert was counted, and the results were expressed as the mean ± standard deviation (SD) from at least three independent experiments.

#### Effect of IL-33 and ST-2 on α-SMA expression in hepatic stellate cells

Hepatic stellate cells (LX-2) were cultured and stabilized in Dulbecco’s modified Eagle medium (DMEM) (cat. no. PM150210, Procell Life Science & Technology Co., Ltd., Wuhan, Hubei, China) prior to intervention. Cells were then digested, resuspended, and seeded into five culture dishes. After a 24-h incubation period, the following treatments were applied: E.m-Ag was added to the positive control dish, while the blank group received no treatment. The remaining dishes were assigned to the eosinophil intervention group, IL-33^−^ adenovirus intervention group, and eosinophil + IL-33^−^ adenovirus intervention group. After 72 h, culture supernatants and suspended eosinophils were removed, and the adherent cells were washed three times with PBS. The adherent HSCs were digested and collected into flow cytometry tubes. An α-SMA primary antibody (smooth muscle actin-specific recombinant antibody, Proteintech cat. no. 14395-1-AP, Proteintech, Rosemont, IL, USA) was added and incubated at room temperature for 30 min, followed by incubation with a fluorescent secondary antibody for 20 min. α-SMA expression was quantified using a BD FACSCelesta flow cytometer (Becton, Dickinson and Company, Franklin Lakes, NJ, USA). This experiment was independently repeated three times.

Through a combination of in vitro and in vivo experiments, we investigated the expression of IL‑33 in the AE lesion microenvironment and its relationship with eosinophils and elucidated the role of IL‑33 in fibrosis within the AE lesion microenvironment. To further explore whether blockade of IL‑33 expression could alter the fibrotic progression of lesions and thereby enhance drug penetration into the lesion core to exert its antiparasitic effect, we subsequently conducted pharmacodynamic experiments involving immunointervention combined with albendazole treatment.

### Intervention in the IL-33/ST-2 signaling pathway combined with ABZ treatment in vivo

#### Validation and screening of IL-33- and ST-2-carrying adenoviruses for intervention

Adenoviral vectors IL-33 and ST-2 genes were constructed by Cyagen Biosciences Inc. (Santa Clara, CA, USA). Adenoviral targeting validation: experimental group (empty vector virus) and control group (saline) were established respectively. A total of 200 μL of adenovirus solution (1 × 10^11^ GC) was administered intravenously for the experimental group via the tail vein to blank mice (*n* = 3), and the control group received an equal volume of saline. Fluorescence imaging was performed 48 h post injection to confirm hepatic localization and viral expression. Screening of gene-carrying adenoviruses: Screening of adenoviruses carrying the IL-33 gene (divided into IL-33 1#, IL-33 2#, and IL-33 3# groups, *n* = 5) and those carrying the ST-2 gene (ST-2 1#, ST-2 2#, and ST-2 3# groups, *n* = 5). Subsequently, additional naive mice received tail vein injections of adenoviruses encoding IL-33 (1 × 10^11^ GC, 200 μL) or ST-2 (1 × 10^11^ GC, 200 μL). After 1 week, hepatic expression levels of IL-33 and ST-2 were assessed using western blotting and quantitative polymerase chain reaction (qPCR) to identify the adenoviruses that induced the highest gene expression. The qPCR primer sequences were as follows: *IL-33* (forward: 5′-CTACTGCATGAGACTCCGTTCTG-3′; reverse: 5′-AGAATCCCGTGGATAGGCAGAG-3′), *ST-2* (forward: 5′-GTGATAGTCTTAAAAGTGTTCTGG-3′; reverse: 5’-TCAAAAGTGTTTCAGGTCTAAGCA-3’), and *β-actin* (forward: 5’-CATTGCTGACAGGATGCAGAAGG-3’; reverse: 5′-TGCTGGAAGGTGGACAGTGAGG-3′). Western blot analyses were conducted to quantify protein expression, with band intensity measured using National Institutes of Health (NIH) ImageJ software (version 1.53; National Institutes of Health, Bethesda, MD, USA). β-actin served as a loading control. The primary antibodies used included anti-ST-2 antibody (BSA-free, ab25877, dilution 1:2000, Abcam, Cambridge, UK) and anti-IL-33 antibody (ab207737, dilution 1:1000, Abcam, Cambridge, UK). All antibodies are presented in Supplementary Table S1.

The validated adenoviral vectors were subsequently administered via tail vein injection into the established echinococcosis model mice, in conjunction with albendazole treatment, to evaluate the therapeutic efficacy of IL‑33 intervention combined with albendazole therapy.

#### Enzyme-linked immunosorbent assay (ELISA) for cytokine quantification in the intervention of the AE model

Venous blood was collected from mice via orbital sinus puncture under anticoagulated conditions. Samples were centrifuged at 4500 g/min for 15 min to separate the serum, which was then stored at −80 °C until analysis. Serum levels of interferon gamma (IFN-γ), interleukin (IL)-4, IL-5, IL-6, and IL-13 were quantified using ELISA kits according to the manufacturers’ instructions. The following kits (Elabscience, Wuhan, Hubei, China) were used: mouse IFN-γ ELISA kit (E-EL-M0048c), mouse IL-4 ELISA kit (E-EL-M0043c), mouse IL-5 ELISA kit (E-EL-M0722c), mouse IL-6 ELISA kit (E-EL-M0044c), and mouse IL-13 ELISA kit (E-EL-M0727c).

#### Western blot analysis of α-SMA expression in the intervention of the AE model

Total protein was extracted from mouse liver tissue and adjusted to a concentration of 3 mg/μL. Equal amounts of protein (15 μg per sample) were separated by 10% sodium dodecyl sulfate–polyacrylamide gel electrophoresis and transferred onto polyvinylidene difluoride membranes. Membranes were blocked with 10% nonfat milk in PBS containing 0.1% Tween-20 for 1 h at 37 °C and subsequently incubated with primary antibodies overnight at 4 °C. After washing three times with PBS containing 0.1% Tween-20, the membranes were incubated with horseradish peroxidase-conjugated secondary antibodies (concentration:1:10,000, cat. no. C31460100, Thermo Fisher, MA, USA) for 90 min at 37 °C. Protein bands were visualized using a Fluor-S MultiImager (Bio-Rad, Hercules, CA, USA). Band densities were quantified using NIH ImageJ software (version 1.53; NIH, Bethesda, MD, USA), with β-actin serving as a loading control. The antibody used was anti-alpha smooth muscle actin antibody (concentration: 1:1500, ab5694, Abcam, Cambridge, UK).

#### Quantitative real-time PCR for α-SMA mRNA expression in the intervention of the AE model

Total RNA was extracted from mouse liver tissues using the RNAprep Pure Animal Tissue Total RNA Extraction Kit (DP431, Tiangen, Beijing, China). Reverse transcription and amplification were performed using the FastKing One-step RT-qPCR kit with SYBR Green detection chemistry (FP313) following the manufacturer’s protocol. Primer sequences were as follows: *α-SMA* (forward, 5′-AGCTACCGAGCCCTGAGTTA-3′; reverse, 5′-TGTTAGAGTGAACGGCCAGC-3′), and *β-actin* (forward, 5′-CATTGCTGACAGGATGCAGAAGG-3′; reverse, 5′-TGCTGGAAGGTGGACAGTGAGG-3′).

#### Masson’s trichrome staining for fibrosis assessment in the intervention of the AE model

Liver tissues were processed, dewaxed, and rehydrated according to the procedures described in section 2.4.1 (Immunohistochemistry). Fibrotic changes were evaluated using a Masson’s trichrome staining kit (cat. no. G1340, Solarbio, Beijing, China). Tissue sections were stained with Weigert’s iron hematoxylin for 5 min, differentiated with acidic ethanol for 10 s, and subsequently stained with Masson’s blue solution for 3 min, Ponceau magenta for 5 min, and aniline blue for 1–2 min. Each staining step was followed by a water rinse. Sections were dehydrated through an ethanol gradient, cleared in xylene, sealed with neutral gum, and imaged microscopically. Quantitative analysis using ImageJ software (version 1.53; NIH, Bethesda, MD, USA, https://imagej.nih.gov/ij/) allowed for the identification, isolation, and extraction of collagen fibers and tissue, from which the collagen volume fraction (CVF) was calculated.

#### Liver and spleen index calculation in the intervention of the AE model

Body weights were recorded prior to euthanasia. Following euthanasia, livers and spleens were excised, blotted dry, and weighed separately. The liver and spleen indices were calculated as a percentage of total body weight using the formula:

$${\mathrm{Organ}}\,{\mathrm{index}}\,\left( \% \right)\, = \,\left( {{{{\mathrm{organ}}\,{\mathrm{weight}}} \mathord{\left/ {\vphantom {{{\mathrm{organ}}\,{\mathrm{weight}}} {{\mathrm{body}}\,{\mathrm{weight}}}}} \right. \kern-0pt} {{\mathrm{body}}\,{\mathrm{weight}}}}} \right)\, \times \,{1}00\,.$$

#### Evaluation of liver function in the intervention of the AE model

Hepatic function was assessed by quantifying serum biochemical markers. These included aspartate aminotransferase (AST), alanine aminotransferase (ALT), total bilirubin (TBIL), conjugated bilirubin (DBIL), alkaline phosphatase (ALP), and γ-glutamyl transpeptidase (γ-GT).

### Statistical analysis

Immunohistochemical data were analyzed using the TissueGnostic Analysis System (version 7.1; StrataQuest, Austria, https://tissuegnostics.com) following panoramic scanning. Quantitative analysis of western blot and Masson’s trichrome staining was performed using ImageJ (version 1.53; NIH, Bethesda, MD, USA, https://imagej.nih.gov/ij/). All data are expressed as the mean ± SD. Statistical analyses were performed using GraphPad Prism software (version 8.2.1; GraphPad, Inc., La Jolla, CA, USA, https://www.graphpad.com). Comparisons between two groups were conducted using Student’s *t*-test, while one-way analysis of variance (ANOVA) was applied for comparisons among multiple groups. A significance level of *α* = 0.05 was considered statistically significant.

## Results

### In vivo elevated IL‑33 and ST‑2 expression in tissues adjacent to hepatic lesions in AE mice

**Fig. 1:**
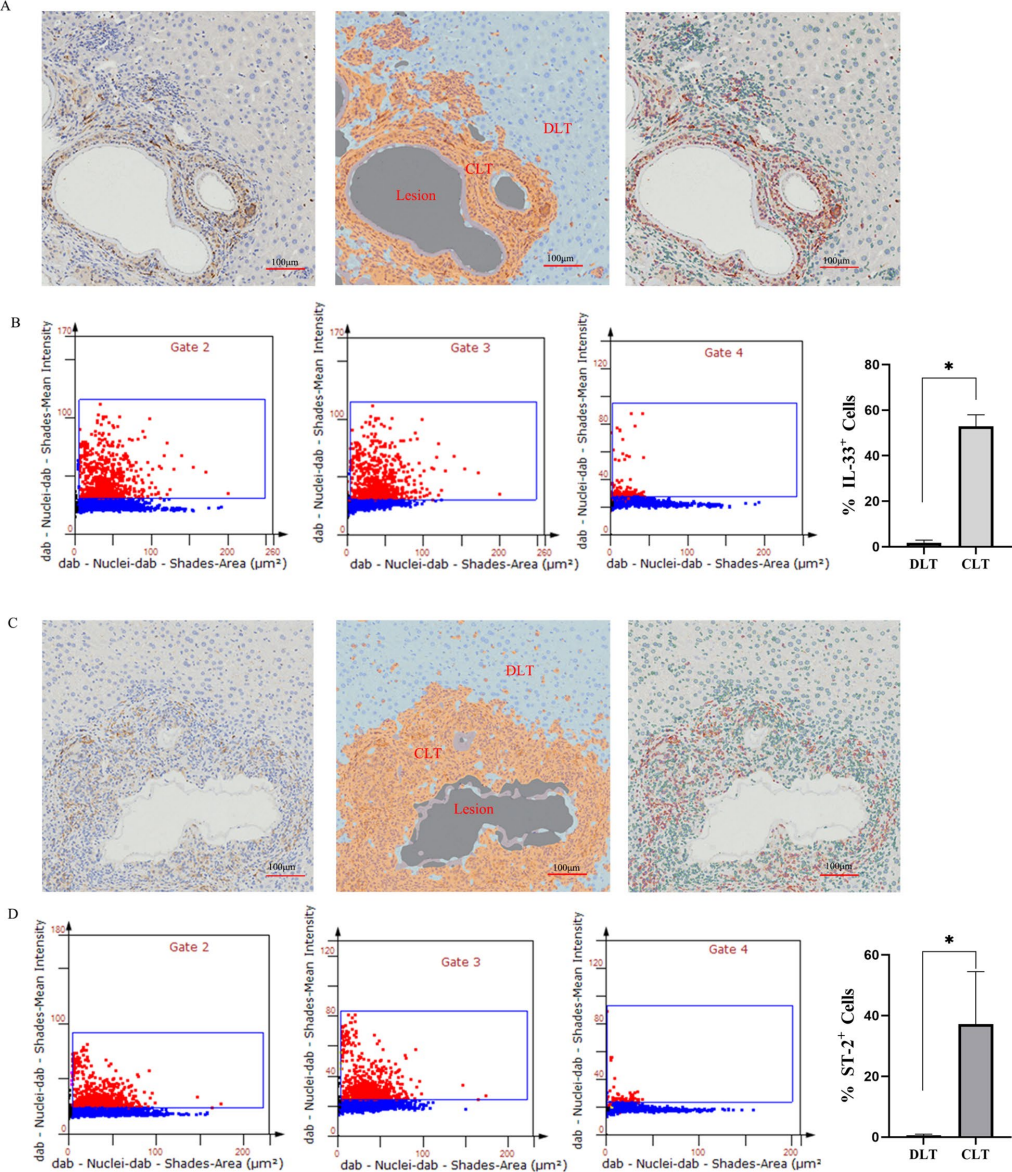
IL-33 and ST-2 expression in hepatic tissue of the AE mouse model. **A**, **C** Immunohistochemical analysis of IL-33 and ST-2 expression in hepatic tissue from mice infected with *E. multilocularis* (*n* = 5). Quantification was performed using the  TissueGnostic Analysis System (StrataQuest, TissueGnostics GmbH, Vienna, Austria). The system-generated schematic delineates lesion areas (gray), close-to-lesion tissue (CLT; yellow), and distant liver tissue (DLT; blue). Positive cells (red) and negative cells (green) were automatically identified. **B**, **D** Flow cytometry-based quantification of IL-33^+^ and ST-2^+^ cells. Gating strategy: Gate 2 denotes all positive cells within the full visual field; gate 3 represents positive cells within the marginal area (CLT); and gate 4 represents positive cells within the normal tissue (DLT). Data are expressed as *mean* ± *SD*. ^*^P < 0.05; no significant difference (ns), *P* > 0.05

Immunohistochemical analysis was conducted to assess IL-33 and ST-2 expression in hepatic tissues of mice with AE. Image analysis was performed using the TissueGnostic Analysis System (StrataQuest, Vienna, Austria). Lesion areas (gray), lesion-adjacent tissue, designated as close-to-lesion tissue (CLT; yellow), and distant liver tissue (DLT; blue) were clearly identified (Fig. 1). The system also identified IL-33- and ST-2-positive cells (red) and negative cells (green). Quantitative analysis revealed significantly higher percentages of IL-33^+^ and ST-2^+^ cells in CLT compared with DLT (*P* < 0.05), indicating elevated IL-33 and ST-2 expression in the fibrotic regions surrounding lesions.

### In vivo increased IL‑33 and ST‑2 expression in hepatic lymphocytes of AE mice

**Fig. 2:**
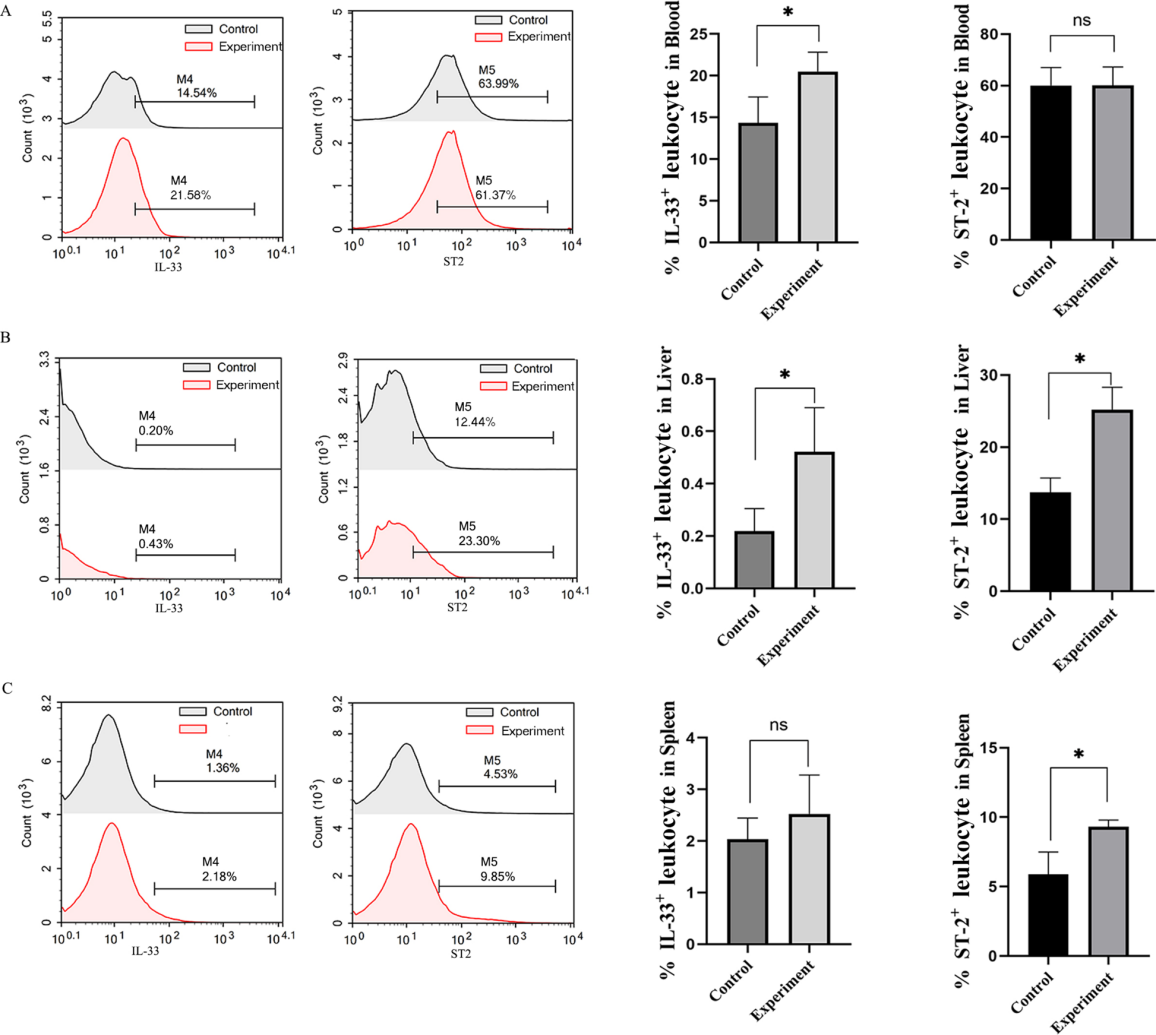
Expression of IL-33 and ST-2 in lymphocytes from the AE mouse model. Flow cytometric analysis of IL-33^+^ and ST-2^+^ lymphocytes in **A** peripheral blood; **B** Hepatic tissue; and **C** Spleen tissue of AE model mice (*n* = 5). Data are presented as *mean* ± *SD*. ^*^P < 0.05; ns, *P* > 0.05

Given the immunoregulatory role of IL-33 and its broad expression in lymphocyte populations, flow cytometric analysis was conducted to assess IL-33 and ST-2 expression in lymphocytes isolated from the peripheral blood (Fig. 2A), liver (Fig. 2B), and spleen (Fig. 2C) of AE model mice. A significant increase in IL-33^+^ lymphocytes was observed in the blood and liver (*P* < 0.05) but not in the spleen. Although ST-2^+^ lymphocyte frequency did not significantly increase in peripheral blood, elevated levels were detected in both liver and spleen tissues (*P* < 0.05). These findings indicate that echinococcosis predominantly induces IL-33 and ST-2 expression in hepatic lymphocytes.

### In vivo upregulation of IL-33 and ST-2 expression in eosinophils of AE mice

**Fig. 3:**
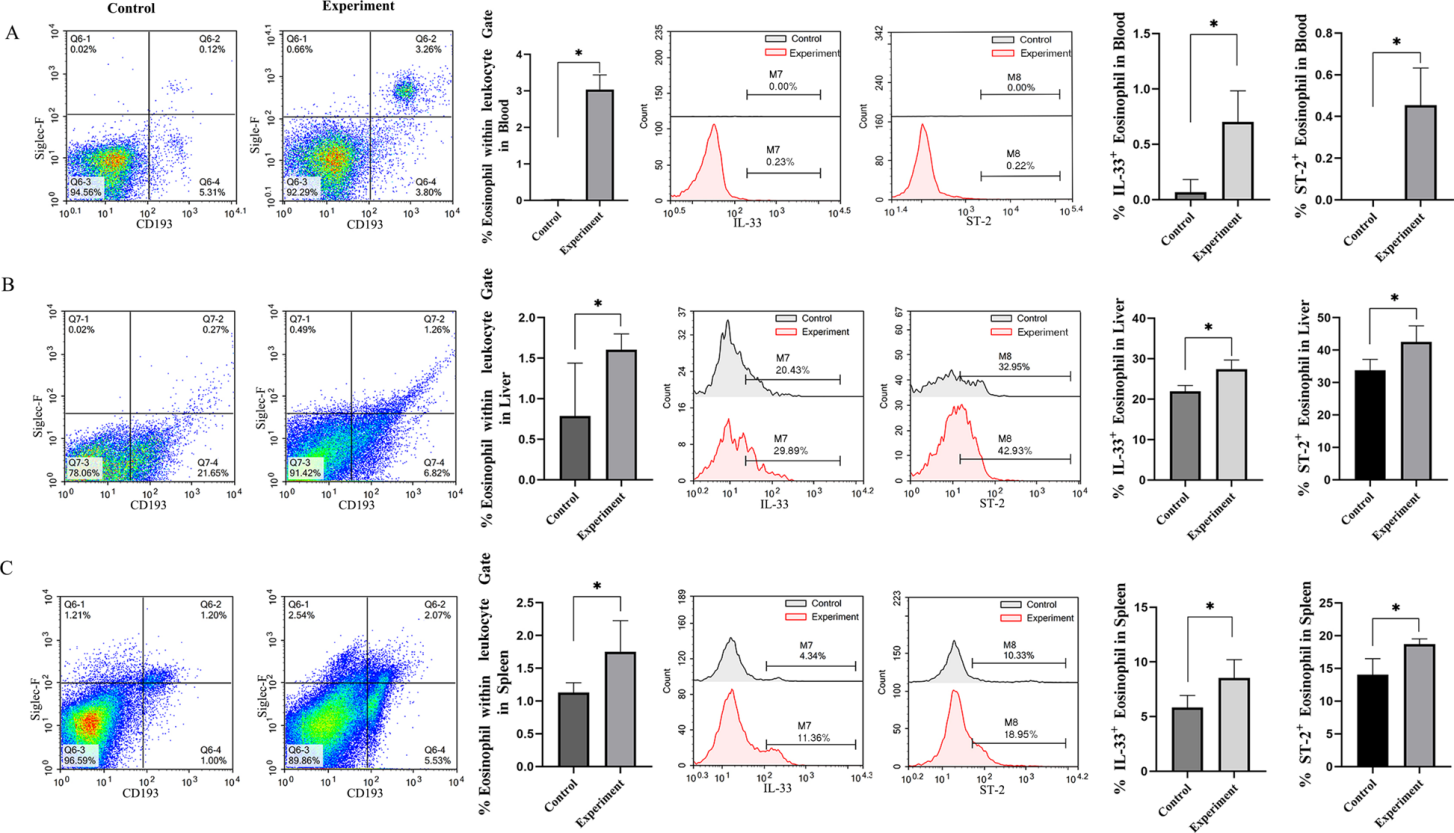
IL-33 and ST-2 expression in eosinophils from the AE mouse model. Flow cytometry was used to quantify IL-33^+^ and ST-2^+^ eosinophils in **A** peripheral blood; **B** hepatic tissue; and **C** spleen tissue of AE model mice (*n* = 5). Data are presented as *mean* ± *SD*. ^*^P < 0.05; ns, *P* > 0.05

Considering the well-documented eosinophilic response to parasitic infection and the role of ST-2 as a key receptor on eosinophil surfaces, IL-33 and ST-2 expression was further analyzed in these cells. AE model mice exhibited higher eosinophil counts in the blood, liver, and spleen than the controls (*P* < 0.05) (Fig. 3A–C). Furthermore, the proportions of IL-33^+^ and ST-2^+^ eosinophils were significantly higher across all three tissues compared with controls (*P* < 0.05). These results indicate that eosinophils play an important role in AE pathophysiology and that IL-33 may regulate eosinophil activity during infection.

### In vitro IL‑33 and ST‑2 promote α‑SMA expression and modulate eosinophil function

**Fig. 4:**
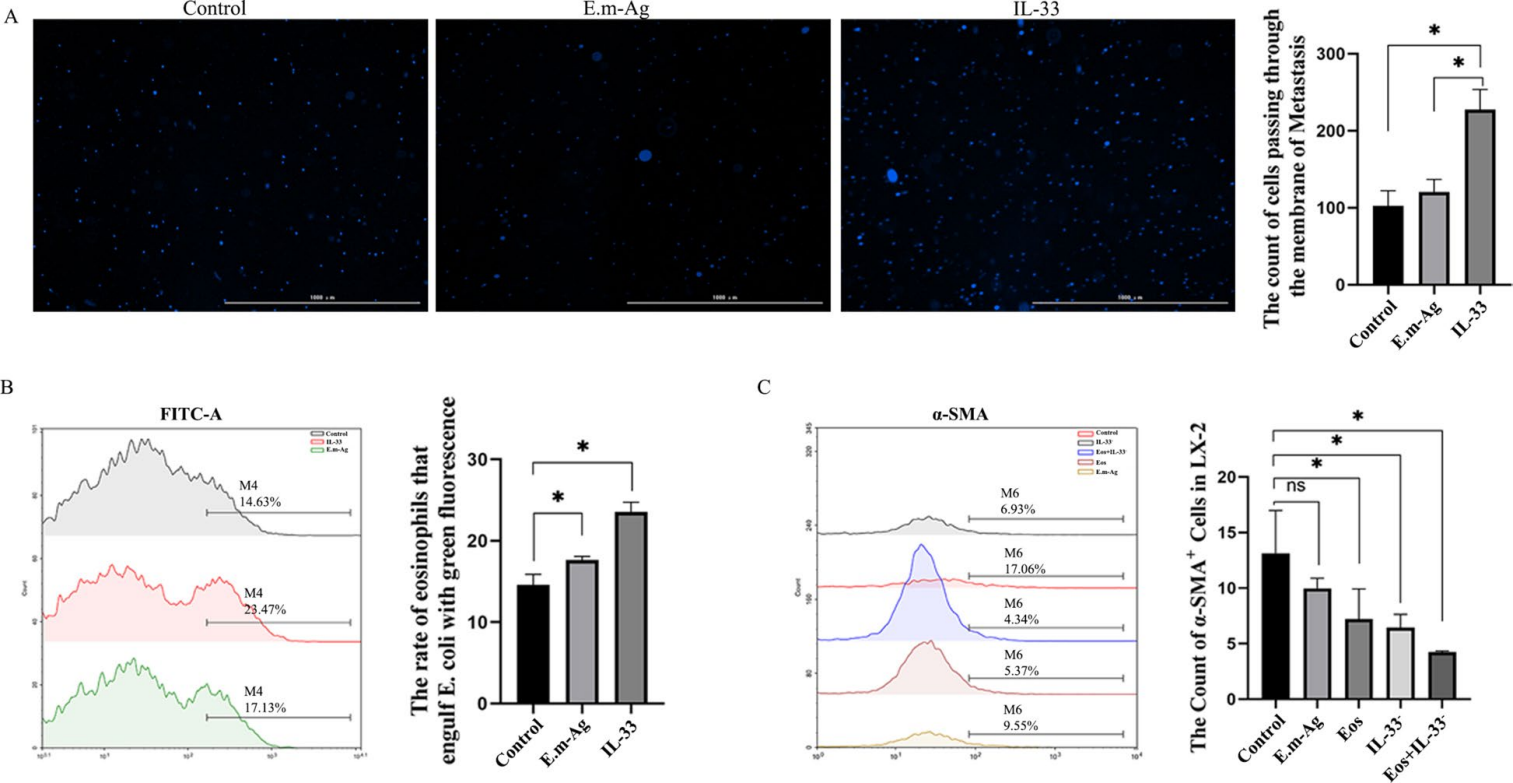
IL-33 and ST-2 enhance α-SMA expression and modulate eosinophil function in vitro. **A** Representative DAPI-stained images from Transwell migration assay comparing control group (*n* = 5), E.m-Ag group (*n* = 5), and IL-33 intervention group (*n* = 5). **B** Flow cytometric assessment of eosinophil phagocytosis of *E. coli* (green fluorescence). **C** Flow cytometric analysis of α-SMA expression in hepatic stellate cells following co-culture and IL-33 intervention. Data are expressed as mean ± SD. ^*^P < 0.05; ns, *P* > 0.05

To further examine the regulatory role of IL-33 in eosinophil function, in vitro experiments were performed. Stimulation of eosinophils with recombinant IL-33 significantly enhanced their migratory capacity, as evidenced by an increased number of cells traversing the Transwell membrane (*P* < 0.05) (Fig. 4A), and also elevated their phagocytic activity against *E. coli* (*P* < 0.05) (Fig. 4B). These results demonstrate that IL-33 promotes both chemotaxis and phagocytosis in eosinophils.

Given the known role of IL-33 in hepatic fibrogenesis, its effect on HSCs was evaluated by measuring α-SMA expression. Suppression of IL-33 in HSCs significantly reduced α-SMA expression (*P* < 0.05), with a greater reduction observed when eosinophils were co-cultured (*P* < 0.05) (Fig. 4C). These findings indicate that IL-33 mediates eosinophil-driven activation of HSCs, thereby contributing to hepatic fibrotic responses.

### In vivo intervention targeting IL-33 and ST-2 combined with ABZ therapy in AE mice

#### Screening and validation of adenoviral vectors for IL-33 and ST-2 silencing

**Fig. 5:**
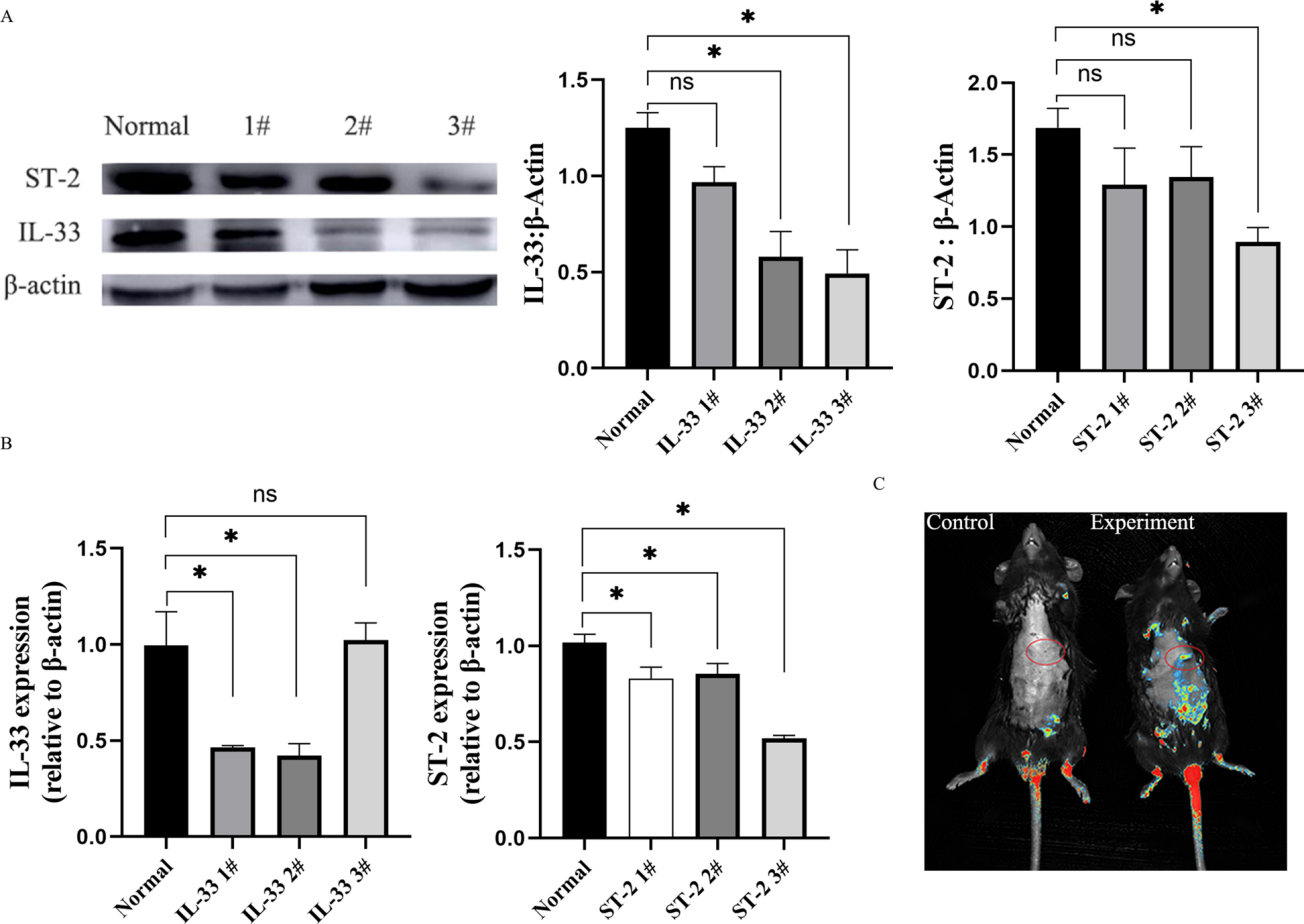
Screening of adenoviral constructs for IL-33 and ST-2 knockdown. **A** Western blot analysis of IL-33 and ST-2 expression in mice transduced with various adenoviral constructs (*n* = 5). **B** qPCR analysis of IL-33 and ST-2 messenger RNA (mRNA) expression levels corresponding to each construct (*n* = 5). **C** In vivo fluorescence imaging confirming hepatic localization of adenoviral vectors following tail vein injection (*n* = 3). Data are expressed as mean ± SD. *P < 0.05; ns, *P* > 0.05

Previous pharmacological studies have indicated that the fibrous capsule surrounding *Echinococcus* lesions impedes drug penetration into the cystic core, thereby diminishing therapeutic efficacy. In the present study, IL-33 was found to mediate eosinophil-regulated expression of α-SMA in HSCs, indicating its potential role in modulating fibrotic processes. To enhance drug delivery and efficacy, the fibrotic response was targeted by silencing the IL-33/ST-2 signaling axis in combination with ABZ treatment, aiming to facilitate deeper drug penetration into parasitic lesions. Low-expression adenoviral vectors targeting IL-33 and ST-2 were constructed by Cyagen Biosciences (Guangzhou, China). Western blot analysis confirmed that all adenoviruses exhibited reduced IL-33 expression (Fig. 5A). Quantitative analysis demonstrated that IL-33 2# and IL-33 3# significantly decreased expression levels, with IL-33 2# identified as the optimal vector on the basis of the combined western blot and qPCR data (Fig. 5B). Similarly, ST-2 3# was identified as the most effective adenovirus for *ST-2* silencing through comparison of western blot and qPCR results (Fig. 5A and B). Following tail vein injection, small-animal live imaging confirmed successful hepatic delivery, with high levels of protein enrichment localized to the liver (Fig. 5C).

#### Masson’s trichrome staining and liver index evaluation

**Fig. 6:**
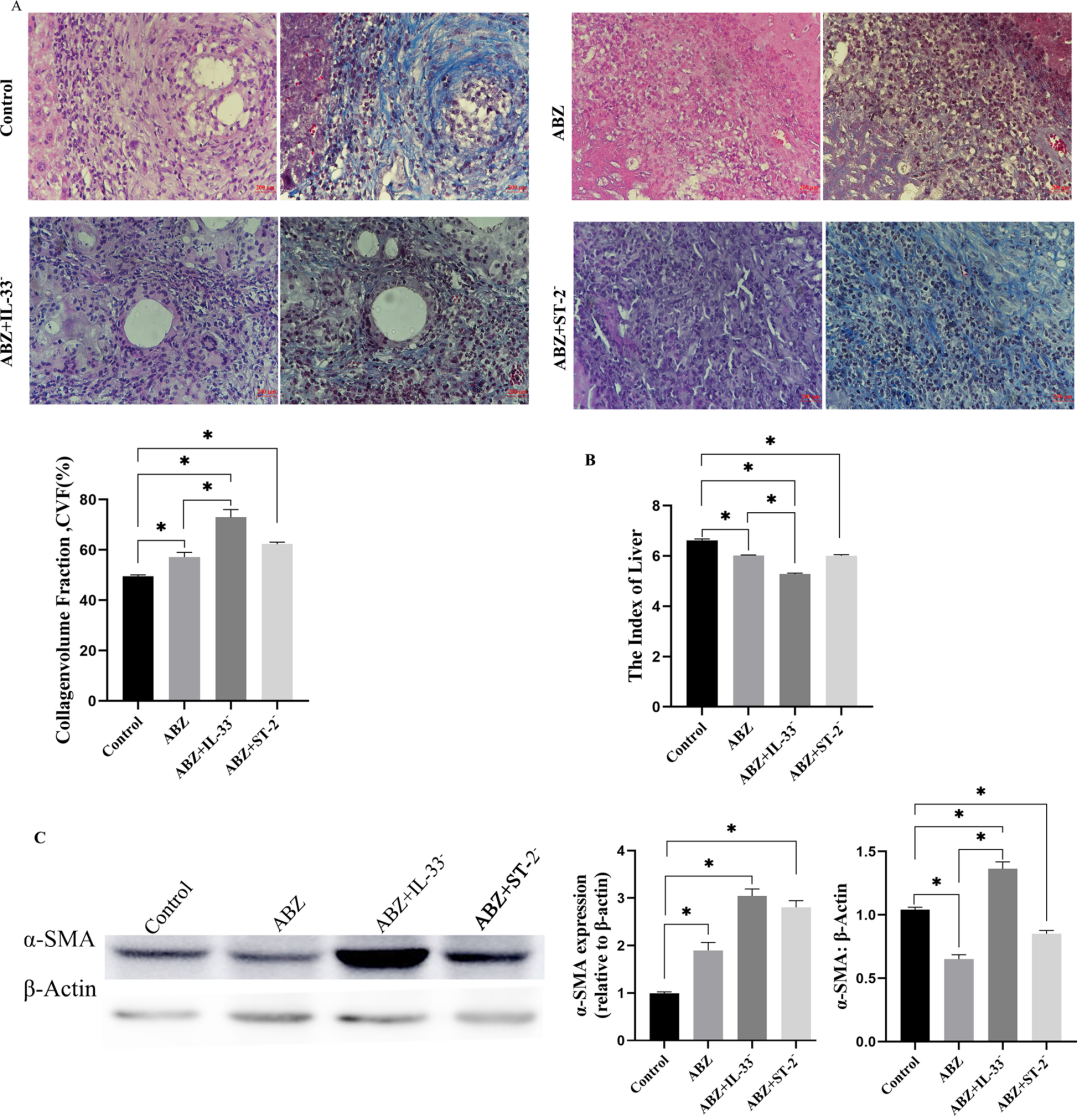
Evaluation of hepatic fibrosis, liver index, and α-SMA expression following treatment. **A** Representative hematoxylin and eosin (H&E) and Masson’s trichrome-stained liver sections from the control, ABZ, ABZ + IL-33^−^, and ABZ + ST-2^−^ groups (*n* = 5 per group). Collagen volume fraction (CVF) was quantified using ImageJ software. **B** Liver index comparison across groups. **C** Western blot analysis of α-SMA protein expression and qPCR quantification of α-SMA mRNA levels. Data are presented as mean ± SD. ^*^P < 0.05; ns, *P* > 0.05

Combined inhibition of IL-33 and ST-2 signaling pathways with ABZ administration produced notable changes in hepatic fibrosis. Masson’s trichrome staining demonstrated a significant increase in CVF in the ABZ-treated group compared with the control group (*P* < 0.05). The ABZ + IL-33^−^ group demonstrated an even greater increase in CVF compared with the ABZ-only group (*P* < 0.05) (Fig. 6A). Western blot analysis further confirmed these findings, revealing significantly elevated α-SMA expression in the ABZ + IL-33^−^ group compared with the ABZ group alone (*P* < 0.05). In contrast, α-SMA levels in the ABZ and ABZ + ST-2^−^ groups were lower than in the control group (*P* < 0.05) (Fig. 6C). Consistent with these findings, qPCR analysis indicated a significant upward trend in α-SMA expression across the experimental groups (*P* < 0.05) (Fig. 6C). Assessment of the spleen index indicated a significant reduction post treatment, with the most pronounced decrease observed in the ABZ + IL-33^−^ group compared with the ABZ group (*P* < 0.05) (Fig. 6). The observed increase in perilesional fibrosis and associated calcification surrounding the lesion, along with a concurrent reduction in the liver index, indicated a relative decrease in lesion size. Collectively, these findings indicate an improved therapeutic outcome following IL-33 pathway intervention.

#### Cytokine profiling by ELISA and spleen index assessment

**Fig. 7:**
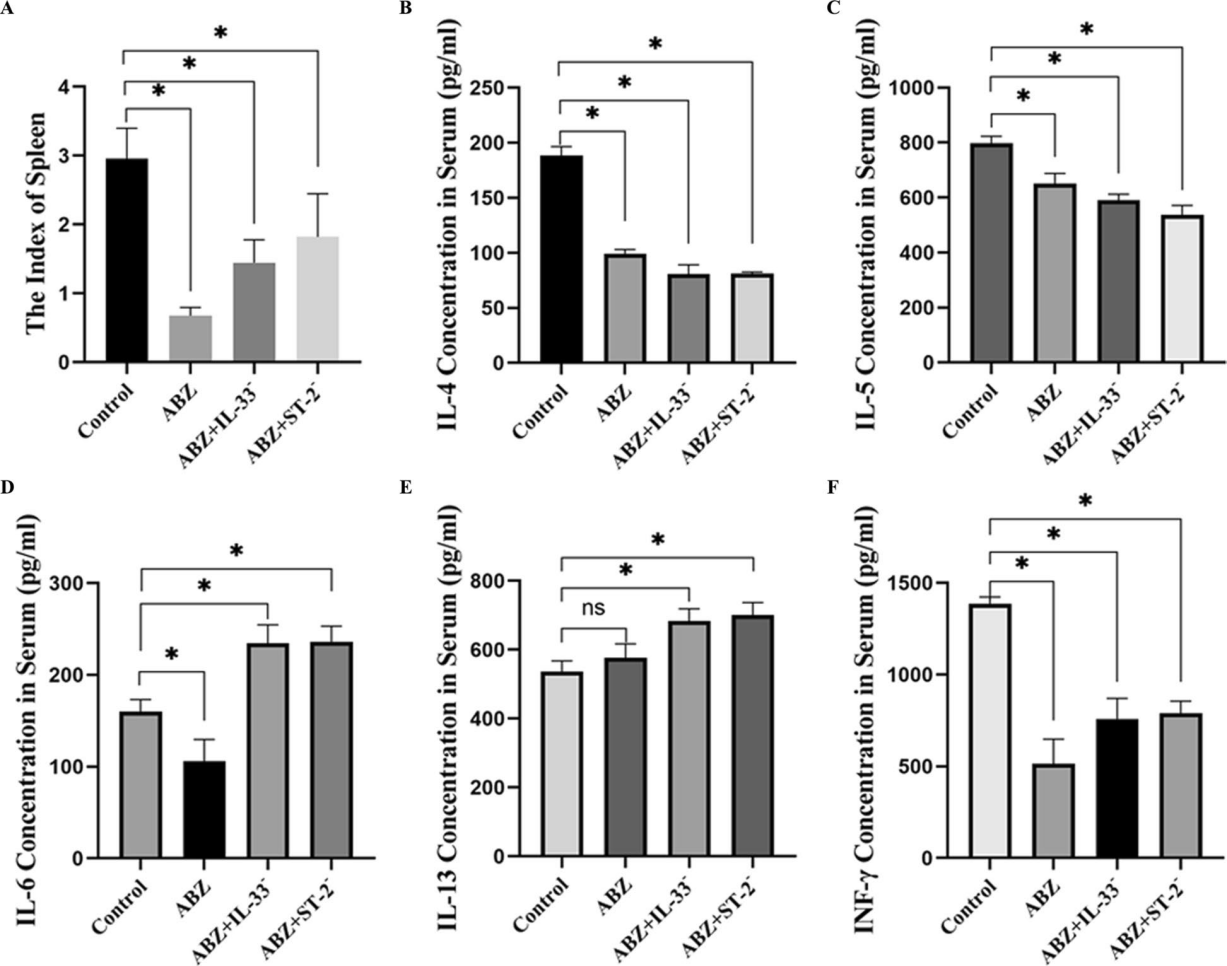
Serum cytokine levels and spleen index following treatment. **A** Comparison of spleen indices among treatment groups (*n* = 5). **B**–**F** Serum levels of IL-4, IL-5, IL-6, IL-13, and IFN-γ determined by ELISA. Data are presented as mean ± SD. ^*^P < 0.05; ns, *P* > 0.05

Spleen index measurements showed a significant decrease following ABZ treatment (*P* < 0.05) (Fig. 7A). ELISA results demonstrated that serum levels of IL-4 and IL-5 were significantly reduced compared with those in the control group (*P* < 0.05) (Fig. 7B and C). IL-6 levels in the ABZ group were lower than those in the control group, whereas the ABZ + IL-33^−^ and ABZ + ST-2^−^ groups exhibited significantly higher IL-6 levels (*P* < 0.05) (Fig. 7D). IL-13 levels increased in both the ABZ + IL-33^−^ and ABZ + ST-2^−^ groups (*P* < 0.05) (Fig. 7E). INF-γ levels decreased significantly compared with those of the control group (*P* < 0.05) (Fig. 7F).

#### Evaluation of liver function

**Fig. 8:**
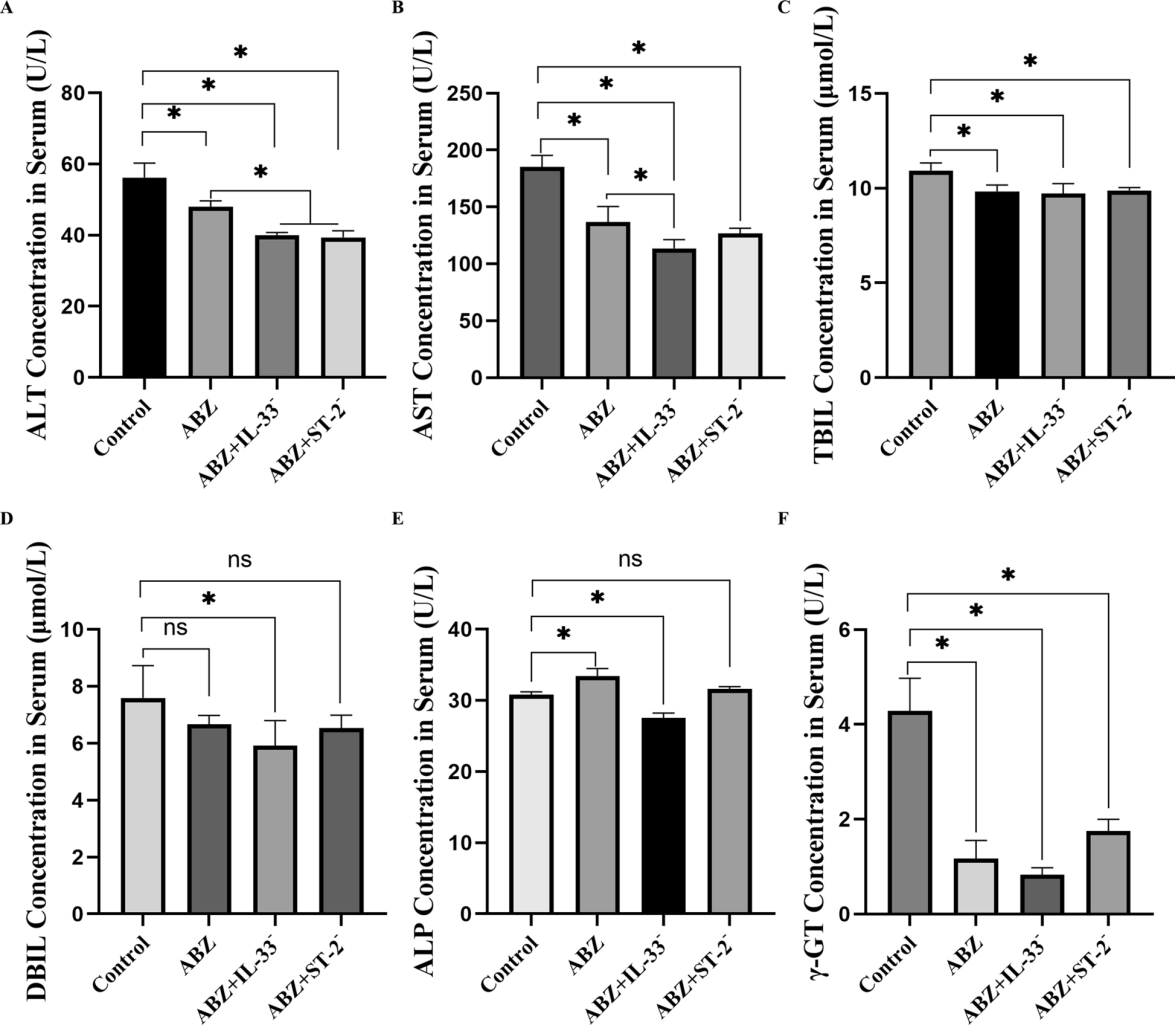
Evaluation of liver function following treatment. Serum biochemical markers assessed include: **A** ALT, **B** AST, **C** TBIL, **D** DBIL, **E** ALP, and **F** γ-GT across groups (*n* = 5). Data are expressed as mean ± SD. ^*^P < 0.05; ns, *P* > 0.05

Serum biochemical analysis demonstrated that the levels of ALT (Fig. 8A), AST (Fig. 8B), TBIL (Fig. 8C), DBIL (Fig. 8D), and γ-GT (Fig. 8F) in the experimental groups were lower than those in the control group. Notably, ALT and AST levels were significantly lower in the ABZ + IL-33^−^ group than in the ABZ group, while ALT levels were also lower in the ABZ + ST-2^−^ group compared with the ABZ group (*P* < 0.05). ALP expression was higher in the ABZ group than in the control group but was reduced in the ABZ + IL-33^−^ group (*P* < 0.05) (Fig. 8). These results collectively indicated that liver function improved following IL-33 and ST-2 pathway intervention combined with ABZ treatment.

## Discussion

Echinococcosis remains highly prevalent in pastoral regions globally, posing a serious threat to public health and adversely affecting both the safety and quality of life of affected populations [25–27]. Current treatment strategies, including both pharmacological and surgical approaches, remain limited in their effectiveness [28, 29]. Pharmacological therapy alone often yields suboptimal outcomes and is associated with adverse effects, while surgical intervention carries a high risk of recurrence [28, 30, 31]. Consequently, there is an urgent, unmet clinical need for novel therapeutic strategies that can overcome this fibrotic barrier, and therapeutic approaches integrating immunomodulation with pharmacologic treatment have been proposed as potentially more effective alternatives. While the IL-33/ST-2 axis is recognized as a key driver of fibrosis in other contexts [15, 32], a critical knowledge gap exists regarding its role and cellular effectors within the unique microenvironment of an active *Echinococcus* lesion. The present study was therefore designed to systematically investigate the role of the IL-33/ST-2 pathway in the hepatic fibrotic response of AE and to explore a novel combination therapy aimed at addressing this critical barrier of drug-impermeable fibrosis.

Immunohistochemical analysis of liver tissues from the murine echinococcosis model indicated that the expression of IL-33 and its receptor ST-2 was markedly elevated at the lesion margins compared with normal hepatic regions, indicating a key role for IL-33/ST-2 signaling in the pathogenesis of echinococcosis. In our preliminary studies, we observed that abnormal collagen fiber expression in the hydatid lesion microenvironment is primarily driven by HSCs [33]; these activated cells, together with ECM components, form the fibrotic capsule that characterizes *Echinococcus* lesions [13, 34].

These findings are consistent with previous research demonstrating a positive association between IL-33 expression and fibrosis severity. Studies employing IL-33-deficient mice have shown attenuation of fibrotic responses, further supporting IL-33 as a key mediator in hepatic fibrogenesis [13, 15, 32]. Building upon this established foundation, our data extend these correlations to the unique context of echinococcosis, strongly implicating IL-33 in the fibrotic remodeling within its specific microenvironment. IL-33, a member of the IL-1 family (also known as IL1RL1), transduces signals through the ST-2 receptor [35, 36]. ST-2 is predominantly expressed on innate immune cells, including macrophages, group 2 innate lymphoid cells (ILC2s), and eosinophils. IL-33 exerts its immunoregulatory functions primarily via binding to ST-2 [18, 37, 38].

To dissect the cellular players in this localized response, we performed a compartmentalized analysis. Flow cytometric analysis of lymphocytes from blood, liver, and spleen in the mouse model revealed increased proportions of IL-33^+^ and ST-2^+^ lymphocytes in the liver, whereas only IL-33^+^ lymphocytes increased in the blood and only ST-2^+^ lymphocytes increased in the spleen. These findings indicate that immune regulation in echinococcosis predominantly occurs within the hepatic microenvironment, reflecting a localized immunopathological process. Concurrently, analysis of eosinophil populations in the liver, blood, and spleen demonstrated elevated eosinophil counts in AE model mice, accompanied by increased proportions of IL-33^+^ and ST-2^+^ eosinophils compared with controls. This is a pivotal finding, as it positions the eosinophil—a cell characteristically abundant in AE lesions—as a potential central hub in this localized loop. This eosinophilic activation likely reflects the immunostimulatory effects of parasitic antigens and excretory–secretory products released by *Echinococcus* larvae during lesion development, which activate innate immunity and induce eosinophil proliferation and activation [39–41].

Therefore, on the basis of the comprehensive analysis above, we speculate that, unlike in peripheral blood and spleen, the expression of IL‑33 and ST‑2 in hepatic lesions is markedly different, indicating a tissue‑specific activation pattern. This likely reflects the recruitment and activation of eosinophils and other IL‑33‑responsive cells within the hepatic microenvironment, driven by local parasitic antigens and chronic inflammation. In contrast, the relatively uniform expression in blood suggests limited systemic circulation of active IL‑33/ST‑2 complexes under steady‑state conditions, while the spleen—acting as a lymphoid reservoir—may exhibit its expression level in relation to its role in filtering blood‑borne antigens and initiating systemic immune responses. This compartmentalized expression pattern highlights the importance of targeting the IL‑33/ST‑2 pathway in the liver of patients infected with AE for effective intervention, as systemic modulation alone may be insufficient to reverse localized fibrotic progression. Moreover, by detecting IL‑33 and ST‑2 expression on the surface of eosinophils, we found that blood, liver, and spleen in the AE model showed significantly different expression levels compared with the normal group, indicating that eosinophils play an important role in AE infection.

To further elucidate the role of IL-33 in modulating eosinophil function, in vitro assays were conducted. Stimulation of eosinophils with recombinant IL-33 significantly enhanced their phagocytic and migratory capacities, indicating that IL-33 promotes eosinophil activation [41, 42]. Given the central role of HSCs in hepatic fibrogenesis, their interaction with eosinophils was also examined [15]. Co-culture experiments demonstrated that inhibition of IL-33 expression in HSCs led to a marked reduction in α-SMA expression, particularly in the presence of eosinophils. Together, these functional data support a working model of a self-perpetuating pathogenic loop: parasitic antigens activate intrahepatic eosinophils; these activated eosinophils then secrete IL-33, which in turn binds to ST-2 on HSCs, driving their activation and collagen production, thereby reinforcing the fibrotic capsule [19, 43, 44].

Guided by this mechanistic insight, we hypothesized that disrupting this loop could dismantle the fibrotic barrier and enhance drug efficacy. In light of these mechanistic insights and the well-documented limitations of ABZ in treating echinococcosis, the dense fibrotic capsule surrounding the lesion was hypothesized to act as a major barrier to effective drug penetration. Previous studies have estimated that less than 5% of systemically administered ABZ reaches the interior of the cyst, primarily owing to the dense fibrous tissue surrounding the lesion [10, 34, 45]. To overcome this limitation, adenoviral vectors engineered to downregulate with low IL-33 and ST-2 expression were developed to target hepatic tissue and modulate the fibrotic environment, thereby facilitating enhanced drug diffusion. On the basis of the screening process, IL-33 2# and ST-2 3# constructs were selected for subsequent in vivo experiments. Vector administration was followed by albendazole treatment (10 mg/kg/day) initiated 72 h after tail vein injection. Masson’s trichrome staining of hepatic tissue demonstrated significantly increased collagen volume fraction and α-SMA expression in the treatment group compared with the control group. Despite a reduced liver index indicative of perifocal calcification, lesion progression was markedly inhibited, indicating favorable treatment outcomes. Moreover, combined IL-33 or ST-2 suppression with ABZ administration yielded superior therapeutic outcomes compared with IL-33 inhibition alone. Collectively, these findings indicate that IL-33 and ST-2 pathway modulation alters the fibrotic microenvironment, enhances ABZ bioavailability within lesions, and improves overall treatment effectiveness. This successful translation from mechanism to therapy represents a powerful demonstration of a “dual-hit” strategy, simultaneously targeting the host-derived fibrotic niche and the parasitic infection.

Spleen index analysis further demonstrated a significant reduction following treatment, reflecting disease control and alleviation of hypersplenism. ELISA results demonstrated decreased serum levels of IL-4 and IL-5 relative to the control group, consistent with reduced eosinophil activation. IL-5 facilitates eosinophil proliferation, maturation, and recruitment, while IL-4 contributes to eosinophil chemotaxis [46–48]. The observed reduction in both cytokines likely reflects normalization of immune function and a decrease in eosinophil-mediated inflammatory activity following treatment. Elevated serum levels of IL-6 and IL-13 were also detected in the treatment groups, aligning with previous evidence indicating that these cytokines are involved in tissue remodeling and fibrogenesis in various organs, including the liver, bone marrow, and pulmonary tissues [49–51]. The increased fibrosis and α-SMA expression observed histologically in treated animals aligns with the upregulation of these fibrogenic cytokines, suggesting that tissue remodeling processes remained active despite effective antiparasitic therapy.

IFN-γ, a pivotal mediator of macrophage activation and type 1 helper T cell (Th1) responses, plays an essential role in host defense against *Echinococcus* infection. In early-stage AE, elevated Th1 cytokines such as IFN-γ and IL-12 are indicative of an active immune response aimed at controlling parasitic proliferation [52, 53]. However, as the disease progresses, immune responses often shift toward a Th2-dominated profile characterized by increased levels of IL-4, IL-5, and IL-10, alongside decreased expression of Th1 cytokines (IFN-γ, IL-12) [34, 54, 55]. In the current study, post-treatment decreases in IFN-γ levels relative to the control group may reflect a shift toward chronic infection dynamics. Biochemical analyses of hepatic function demonstrated significant reductions in ALP, AST, DBIL, TBIL, and γ-GT across treatment groups. These findings are indicative of improved hepatic function. Notably, the most substantial improvements were observed in mice receiving combined IL-33 pathway inhibition and albendazole therapy, reinforcing the conclusion that suppression of the IL-33/ST-2 axis not only disrupts fibrosis but also provides significant hepatoprotective benefits.

In summary, by systematically linking the IL-33/ST-2 axis to eosinophil and hepatic stellate cell cross-talk, our study makes three pivotal contributions: (A) It identifies a novel pathogenic mechanism in AE wherein a parasitic antigen-driven, IL-33/ST-2-mediated cross-talk between eosinophils and HSCs perpetuates fibrosis; (B) it redefines the role of eosinophils, positioning them as critical upstream regulators of the fibrotic niche; and (C) it provides the first preclinical validation of a mechanism-based combination therapy, demonstrating that targeted pathway inhibition, when combined with albendazole, synergistically enhances therapeutic efficacy compared with monotherapy. These findings offer a potential theoretical and experimental framework for developing novel immunomodulatory strategies to improve the clinical management of echinococcosis, particularly where conventional treatments are insufficient.

Notwithstanding these significant findings, we acknowledge the limitations of our study and have defined clear avenues for future research. First, the initial liver-targeting validation used a limited sample size of only three mice; although the results were consistent, future studies with larger cohorts are required for rigorous statistical validation. Second, our mechanistic exploration utilized the EOL-1 cell line, which, as a non-terminally differentiated model, may not fully recapitulate primary cell function. Future validation of our eosinophil-related findings in primary human or murine cells is therefore essential. Third, as a proof-of-concept study, we did not include clinical specimen analysis. We plan to address this in future work by collecting patient samples to assess the human relevance of our findings through the analysis of key biomarkers.

Nevertheless, this study successfully validates a novel liver-targeting strategy and provides critical evidence for the central role of the IL-33/ST-2 axis in AE-associated fibrosis. We believe that by systematically addressing these limitations, our findings hold significant promise for advancing the understanding of AE immunopathology and for paving the way toward clinical translation.

## Conclusions

This study delineates a novel pathogenic mechanism in AE, whereby a parasitic antigen-driven IL-33/ST-2 axis promotes fibrosis by mediating cross-talk between eosinophils and hepatic stellate cells. This work repositions eosinophils as pivotal upstream architects of the fibrotic niche and provides the first preclinical validation of a synergistic combination therapy that significantly boosts the efficacy of albendazole. Collectively, these findings lay a foundation for developing novel immunomodulatory strategies to improve clinical outcomes for AE.

## Supplementary Information

Supplementary material 1: Figure S1. Gating strategy for flow cytometric analysis.

Supplementary material 2: Table S1. All antibodies.

## Data Availability

Data supporting the main conclusions of this study are included in the manuscript.

## Abbreviations

CE: Cystic echinococcosis
AE: Alveolar echinococcosis
ABZ: Albendazole
DCs: Dendritic cells
CVF: Collagen volume fraction
CLT: Close-to-the-lesion tissue
DLT: Distant-from-the-lesion tissue
HAE: Hepatic alveolar echinococcosis
ECM: Extracellular matrix
Eos: Eosinophil
IHC: Immunohistochemistry
HSC: Hepatic stellate cell
IL: Interleukin
ELISA: Enzyme-linked immunosorbent assay
WB: Western blotting
IF: Immunofluorescence
α-SMA: Alpha-smooth muscle actin
ST-2: Suppression of tumorigenicity 2
ILC2: Type 2 innate lymphoid cells
TNF: Tumor necrosis factor
ALP: Alkaline phosphatase
IFN: Interferon
γ-GT: γ-Glutamyl transpeptidase
DBIL: Direct bilirubin
TGF: Transforming growth factor
TBIL: Total bilirubin
AST: Aspartate aminotransferase
ALT: Alanine transaminase
