# Supplementary figures and images for: Therapeutic effects of IL-33/ST-2 pathway inhibition combined with albendazole on hepatic fibrosis and immune regulation in alveolar echinococcosis: in vivo and in vitro evidence
Source: Parasit Vectors. 2026 Apr 3;19:177. doi: 10.1186/s13071-026-07355-8 (PMC13104343; doi:10.1186/s13071-026-07355-8)

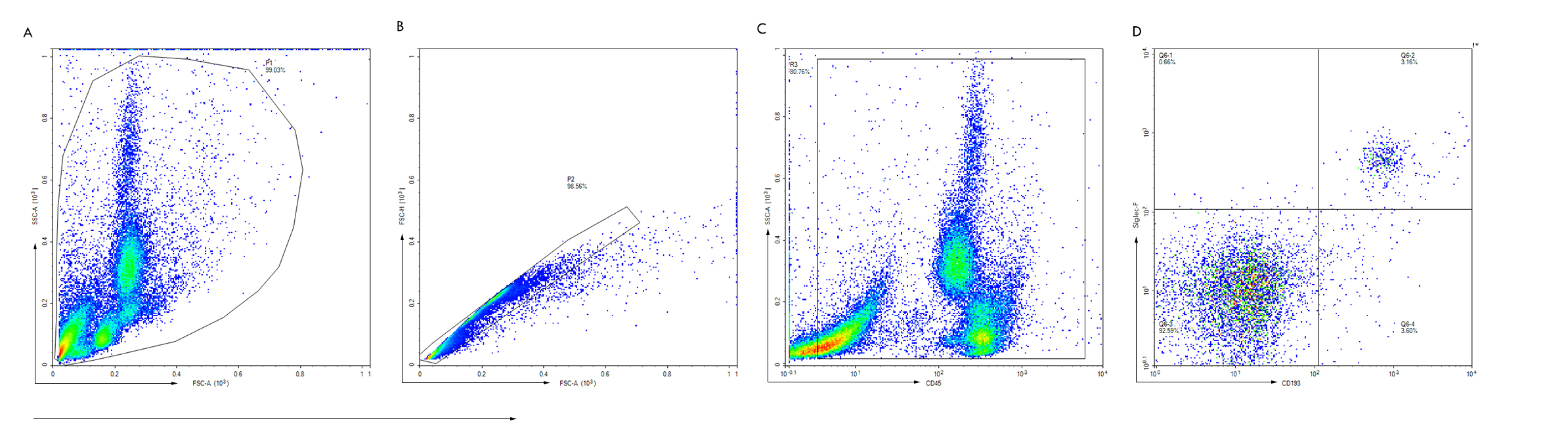

Supplement: Supplementary file 1 — Supplementary material 1: Figure S1. Gating strategy for flow cytometric analysis. [file 13071_2026_7355_MOESM1_ESM.jpg]
