# Supplementary material for: Therapeutic effects of IL-33/ST-2 pathway inhibition combined with albendazole on hepatic fibrosis and immune regulation in alveolar echinococcosis: in vivo and in vitro evidence
Source: Parasit Vectors. 2026 Apr 3;19:177. doi: 10.1186/s13071-026-07355-8 (PMC13104343; doi:10.1186/s13071-026-07355-8)
Supplement: Supplementary file 2 — Supplementary material 2: Table S1. All antibodies. [file 13071_2026_7355_MOESM2_ESM.doc]

**Supplementary Table 1.** All antibodies

| Antibodies | SOURCE | IDENTIFIER |
| --- | --- | --- |
| Smooth muscle actin-specific Recombinant antibody | Proteintech | Cat:14395-1-AP |
| Anti-ST2 antibody | Abcam | Cat: ab194113 |
| Anti-ST2 antibody | Abcam | Cat: ab25877 |
| Anti-IL-33 antibody | Abcam | Cat: ab187060 |
| Anti-IL-33 antibody | Abcam | Cat: ab207734 |
| PE Anti-IL-33 antibody | Abcam | Cat: ab282176 |
| anti-ST2 antibody - BSA free | Abcam | Cat: ab25877 |
| Anti-alpha smooth muscle Actin antibody | Abcam | Cat: ab5694 |
| APC anti-mouse CD45 Recombinant Antibody | Biolegend | Cat: 157606 |
| PerCP/Cyanine5.5 anti-mouse CD193 (CCR3) Antibody | Biolegend | Cat: 144516 |
| FITC anti-mouse CD170 (Siglec-F) Antibody | Biolegend | Cat: 155504 |
| Brilliant Violet 421™ anti-mouse IL-33Rα (IL1RL1, ST2) Antibody | Biolegend | Cat: 145309 |
